# Clinical proof-of-concept trial to assess the therapeutic effect of sirolimus in patients with autosomal dominant polycystic kidney disease: SUISSE ADPKD study

**DOI:** 10.1186/1471-2369-8-13

**Published:** 2007-09-15

**Authors:** Andreas L Serra, Andreas D Kistler, Diane Poster, Marian Struker, Rudolf P Wüthrich, Dominik Weishaupt, Frank Tschirch

**Affiliations:** 1 Clinic for Nephrology, University Hospital, CH-8091 Zürich, Switzerland; 2 Institute of Diagnostic Radiology, University Hospital, CH-8091 Zürich, Switzerland

## Abstract

**Background:**

Currently there is no effective treatment available to retard cyst growth and to prevent the progression to end-stage renal failure in patients with autosomal dominant polycystic kidney disease (ADPKD). Evidence has recently been obtained from animal experiments that activation of the mammalian target of rapamycin (mTOR) signaling pathway plays a crucial role in cyst growth and renal volume expansion, and that the inhibition of mTOR with rapamycin (sirolimus) markedly slows cyst development and renal functional deterioration. Based on these promising results in animals we have designed and initiated the first randomized controlled trial (RCT) to examine the effectiveness, safety and tolerability of sirolimus to retard disease progression in ADPKD.

**Method/design:**

This single center, randomised controlled, open label trial assesses the therapeutic effect, safety and tolerability of the mTOR inhibitor sirolimus (Rapamune^®^) in patients with autosomal dominant polycystic kidney disease and preserved renal function. The primary outcome will be the inhibition of kidney volume growth measured by magnetic resonance imaging (MRI) volumetry. Secondary outcome parameters will be preservation of renal function, safety and tolerability of sirolimus.

**Discussion:**

The results from this proof-of-concept RCT will for the first time show whether treatment with sirolimus effectively retards cyst growth in patients with ADPKD.

**Trial registration:**

NCT00346918

## Background

Autosomal dominant polycystic kidney disease (ADPKD) is the most common hereditary cause of end-stage renal disease (ESRD), affecting all ethnic groups worldwide, with an incidence of 1 in 500 to 1 in 1000 [1]. ADPKD is characterized by the progressive development of innumerable cysts in both kidneys, which distort the normal kidney architecture and leads to a loss of renal function. The development of renal failure is highly variable, but typically patients develop ESRD by the age of 40 to 50 years, necessitating renal replacement therapy (RRT) and/or kidney transplantation [2]. Apart from blood pressure control and symptomatic treatment of cyst bleedings and infections there is no curative therapy for this disease [3]. PKD1 and PKD2 encode the proteins polycystin-1 and polycystin-2 which are expressed in the kidney and function together to regulate growth and morphologic configuration of renal epithelial cells [4]. Mutation in PKD1 leads to a more severe phenotype of ADPKD than mutations in PKD2, with ESRD occurring on average 20 years earlier (53.4 versus 72.7 years) [5].

In ADPKD progressive cyst growth generally precedes the development of renal insufficiency. Compensatory mechanisms (hyperfiltration) maintain renal function virtually normal for decades despite continuous cyst growth. By the time renal function starts to decline, the kidneys are usually grossly enlarged with little normal renal parenchyma recognisable on imaging studies. Data from the consortium for radiologic imaging studies of polycystic kidney disease (CRISP) and others have shown that the rate of kidney volume growth is a predictor of renal functional decline and therefore kidney volume is used as surrogate marker of disease progression especially in clinical intervention trials for ADPKD [6,7]

Non-invasive radiologic methods are available to monitor the growth rate of kidney volume. Renal ultrasound measurements are operator-dependent and not precisely reproducible. Unenhanced and contrast enhanced Computer tomography (CT) scanning is reported to be an accurate method to determine kidney volume, but it involves ionizing radiation and potentially nephrotoxic contrast medium and is therefore not an ideal method in patients with reduced kidney function needing repetitive measurements [8,9]. Due to its high soft tissue contrast and the lack of ionizing irradiation Magnetic resonance imaging (MRI) is also considered useful to monitor kidney volume changes in ADPKD. The analysis of sequential MRI scans was shown to be accurate to monitor rates of kidney volume enlargement in ADPKD [7].

Sirolimus is an immunosuppressant that binds to FK Binding Protein-12 (FKBP-12) and inhibits the activation of the mTOR, a key regulatory kinase of growth and proliferation. Sirolimus is approved for the prevention of graft rejection following renal transplantation. Due to its antiproliferative properties it is also used in coated stents to prevent coronary artery restenosis after angioplasty [10]. Furthermore it has shown clinical effectiveness in kidney transplant recipients with Kaposi's sarcoma [11].

We have shown previously that the mTOR inhibitors rapamycin and everolimus effectively reduce cyst growth and loss of renal function in an experimental animal model for PKD [12,13]. Additional studies have shown that rapamycin is also effective in various mouse models of polycystic kidney disease, including dominant and recessive forms [14]. Of interest, an analysis of ADPKD patients which received a renal transplant, revealed that cystic kidney volumes regressed under immunosuppression with sirolimus [15]. Based on these promising results we have designed and initiated the first clinical trial to examine the effectiveness and safety of sirolimus in young patients with early manifestations of ADPKD and intact renal function.

## Methods/Design

### Study aim

**Figure 1:**
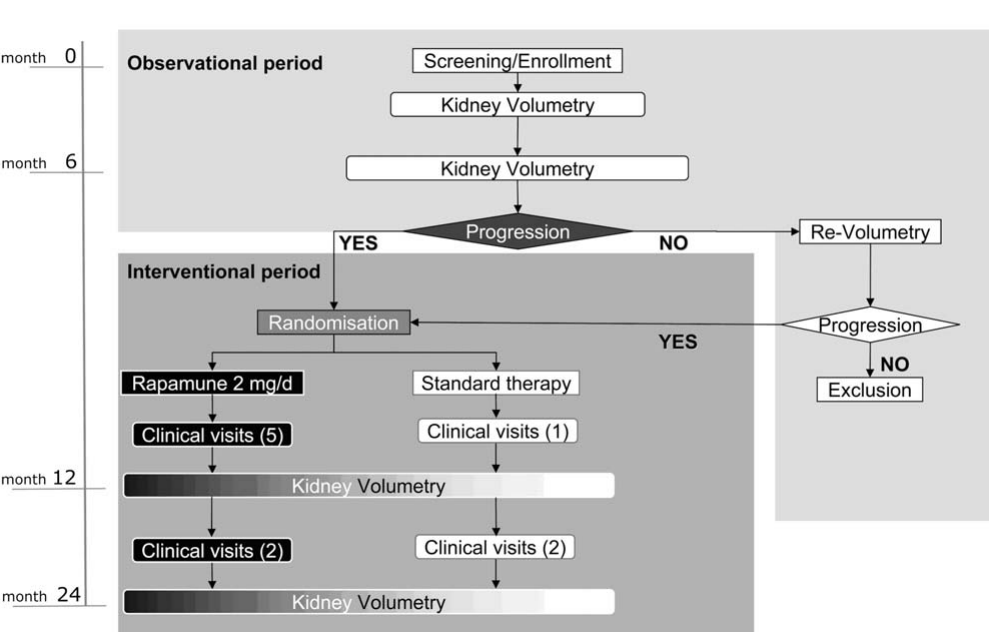
Flow chart of SUISSE ADPKD study.

The primary objective of the SUISSE ADPKD study is to assess the effectiveness of sirolimus to retard kidney volume growth and to prevent the loss of renal function in young patients with ADPKD and preserved renal function. Patients with ADPKD and kidney volume growth that can be documented within 6 months are randomized to treatment with sirolimus 2 mg/day for 18 months (Figure 1) or standard treatment. The secondary objectives are to follow renal function and blood pressure and to monitor for the occurrence of proteinuria. Safety and tolerability of sirolimus treatment in ADPKD patients will also be assessed.

### Study design and setting

The study is a single center randomised controlled open label trial which is open to ADPKD patients with a positive family history of ESRD due to ADPKD thereby selecting mostly patients with mutations in the PKD1 gene. The study will involve 100 ADPKD-patients aged 18–40 years with a creatinine clearance >70 ml/min. Kidney volumes will be measured by MRI without contrast media at study month 0 and 6. Patients with documented volume progression are randomized at a 1:1 ratio to sirolimus 2 mg/day or standard treatment for 18 months. Recruitment has started in May 2006 and will last until December 2007. The study will be completed by December 2009.

### Ethical considerations

Ethical approval has been obtained form the local ethics committee of the University Hospital Zürich.

### Study drug and dosing

Rapamune^® ^(sirolimus) will be used at a fixed dose of 2 mg daily to achieve sirolimus through levels between 4–10 μg/L. By using this rather low dose of sirolimus as monotherapy we expect a low incidence of sirolimus-related adverse events. The dose will be reduced or withheld in case of a through level exceeding 10 μg/l, elevated liver enzymes (> 2-fold above normal values), thrombopenia (< 100'000/mm^3^), leukopenia (< 3'000/mm^3^) or serious sirolimus-associated toxicity.

### Study drug adherence

Adherence to the prescribed study drug will be assessed by using the Medication Event Monitoring V Track-Cap System (MEMS^®^, Aardex, Ltd., Zug, Switzerland) during the complete treatment period. The MEMS^® ^assesses the medication adherence reliably and sensitive as a period with a lack of medication bottle opening documentation, representing most likely an episode of non-adherence [16,17]. The system monitors electronically the date and time of the medication bottle opening. We will measure 1) treatment adherence as the proportion of medication vial caps opened in a given month relative to the prescribed doses for that month, 2) dosing adherence as the percentage of days with correct dosing and 3) drug holidays, as the number of periods without drug intake that exceeded 48 hours.

### Identification of eligible patients

Study participation is offered to all eligible patients with ADPKD. ADPKD patients suffering from advanced renal failure including dialysis patients and transplant recipients treated at our clinic are informed about the study and screening for ADPKD is offered to their relatives. All nephrology clinics and dialysis units in Switzerland have been informed and information material (print and in the internet [18]) for study participants and health professionals has been provided. Potential study participants are invited for a screening visit including medical history, physical examination, renal ultrasonography and blood and urine analyse.

Male or female ADPKD patients aged 18 to 40 years with a creatinine clearance ≥ 70 ml/min are eligible for the study if they exhibit a kidney volume progression over the observational pre-randomisation period of 6 months. The diagnosis of ADPKD is based on ultrasonographic diagnostic criteria in patients with a family history of polycystic kidney disease [19]. In patients with negative family history, proof of a mutation in the PKD1 or PKD 2 genes is required for inclusion (sequencing analysis; Athena Diagnostics, Inc., Worcester, MA, USA). Detailed study inclusion and exclusion criteria are given in table 1 and 2.

**Table 1:** Study inclusion criteria

• Age 18 to 40 years
• GFR ≥ 70 ml/min (Cockcroft - Gault formula)
• Diagnosis of ADPKD:
○ Positive family history for ADPKD
■ patients < 30 years: ≥ 2 cysts in either kidney
■ patients ≥ 30 years: ≥ 2 cysts in each kidney
○ Negative family history for ADPKD but sonographically cystic kidney disease: proof of a mutation in the PKD1 or PKD2 gene is required (Athena Diagnostics, Inc., Worcester, MA, USA)
• Documented kidney volume enlargement (MRI volumetry)
• Signed informed consent

**Table 2:** Study exclusion criteria

• Female patient of childbearing potential who is unwilling to use effective means of contraception
• Increased liver enzymes (2-fold above normal values)
• Hypercholesterolemia (fasting cholesterol > 8 mmol/l) or hypertriglyceridaemia (> 5 mmol/l) not controlled by lipid lowering therapy
• Granulocytopenia (white blood cell < 3,000/mm3) or thrombocytopenia (platelets < 100,000/mm3)
• Infection with hepatitis B or C, HIV
• History of malignancy
• Mental illness that interferes with the patient ability to comply with the protocol
• Drug or alcohol abuse within one year of baseline
• Co-medication with strong inhibitor of CYP3A4 and or P-gp like voriconazole, ketoconazole, diltiazem, verapamil, erythromycin or with a strong CYP3A4 and or P-gp inductor like rifampicin
• Known hypersensitivity to macrolides or Rapamune^®^
• Patients who are unwilling or unable to give informed consent

### Randomization and study blinding

Patients are randomized at a one to one ratio to sirolimus or standard treatment alone. The randomisation list has been generated by a biostatistics unit which is independent of the study team using a permuted blocks design with a random block size of 4 and 6 to guarantee a balanced allocation. The randomisation codes are kept in sealed sequentially numbered opaque envelopes and are not opened until two MR scans within 6 months have shown an enlargement of the total kidney volume of ≥ 2 %.

### Primary outcome

The primary objective of the SUISSE ADPKD study is to determine the effect of sirolimus treatment on kidney volume enlargement in ADPKD patients with preserved renal function. Patients with documented kidney volume growth in the last six months will be randomized to sirolimus or standard treatment. Kidney volume will be measured 6 and 18 months after randomization and the percent annual growth of the combined (left and right) kidney volume will be calculated.

### Secondary outcome

Absolute kidney volume growth from inclusion to month 18 will be assessed as a secondary outcome. Other secondary objectives are to compare blood pressure, renal function and proteinuria in patients with sirolimus or standard treatment and to assess safety and tolerability of sirolimus treatment. New onset or progression of arterial hypertension might reflect disease progression of ADPKD or a potential adverse drug effect and will be assessed comparing the change of blood pressure and the change of antihypertensive drug dosage during follow up. Renal function will be assessed by using estimation equations (Cockcroft-Gault and cystatin C) as well as measured creatinine clearance determined by 24-hour urine collection. Proteinuria will also be measured by 24-hour urine collection. The frequency and severity of all reported adverse events will be recorded, including laboratory abnormalities such as anemia, thrombocytopenia and hyperlipidemia.

### Magnetic resonance imaging

All individuals undergo MR imaging of the kidneys using a 1.5 Tesla scanner. For signal reception in all examinations an 8-channel anteroposterior phased-array surface coil (torso array coil) is placed around the patient and covers the entire kidneys. The imaging protocol includes unenhanced sequences only. In order to get an overview of the extent of the cystic disease of the kidneys a coronal single shot fast spin echo (SSFSE) sequence is acquired in breath hold technique. The MR imaging parameters of this sequence are as follows: repetition time (TR) msec/echo time (TE) msec 1349/90.1; field of view 48 × 48 cm; acquisition matrix 384 × 224; section thickness 4 mm; no interslice gap. Based on this coronal sequence the transaxial sequences are planned. The transaxial sequences consists of two breath hold T1-weighted fast spoiled gradient echo (FSPGR) sequences (TR msec/TE msec = 85/1.4) with two different slice thicknesses (3 and 4 mm, respectively). Other parameters of the T1-weighted gradient-echo pulse (GRE) sequences are: field of view 48 × 48 cm, matrix 256 × 160; no interslice gap. In addition a transaxial T2-weighted fast spin echo (FSE) sequence with respiratory triggering is performed (TR msec/TE msec = 17143/102.8; field of view 48 × 48 cm, matrix 256 × 160, thickness 3 mm; no interslice gap). To measure the kidney volumes the transaxial breath hold T1-weighted FSPGR sequence with a slice thickness of 3 mm is primarily used. In case of a more advanced disease with large polycystic kidneys and/or when respiratory artefacts are present the volume measurements are either performed on the transaxial breath hold T1-weighted FSPGR sequence with a slice thickness of 4 mm or on the transaxial T2-weighted FSE sequence. Once one of these sequences is chosen the same sequence is used for volume measurements in the following MR imaging sessions.

### Renal volume measurements

Two independent trained observers perform a manual segmentation of both kidneys for each patient. To prevent bias, the observers are blinded to all clinical and radiological data, their first measurements and the results of the other observer. The measurements are performed in random order. Blinding is performed with regard to patients and the different time points when imaging has been obtained. Manual segmentation is performed electronically on an interactive workstation (Advantage Windows Workstation; GE Medical Systems Europe, Buc, France). Each kidney is assessed separately. On each section, the outlines of the kidney are manually drawn by using the computer mouse. The vessels and the ureter in the area of the renal hilum are excluded from manual volumetric marking. The volume corresponding to each outline is obtained by multiplying the area of the outline by the section thickness. The total volume of the kidney segments is obtained by summing the volume of each section. The manufacturer's software automatically calculates the total volume after drawing the outlines of the kidney on all sections. Mean values of the measurements performed by the two independent observers will be used for analysis. In case of a large disagreement, both observers will repeat measurement.

### Data collection

Data will be collected into a web-based data base designed to capture all visit information including medical history, results form laboratory analysis and adverse events. Baseline data and ongoing data collection as outlined in table 3 will be obtained.

**Table 3:** Baseline and ongoing data collection

**Baseline data and follow-up data at month 6, 12 and 24**
MRI kidney volumetry
Creatinine clearance and estimated GFR (Cockcroft-Gault)
Proteinuria (24-hour urine and spot urine)
Physical examination and vital signs
Laboratory tests
Haematology/Biochemistry
Lipid profile
Sirolimus trough level
Pregnancy test^1^
Serological testing for hepatitis B, C and human immunodeficiency virus (HIV)^1^
MEMS^® ^check
Adverse events and concomitant therapy

**Follow-up data every 3 months**

Serum creatinine
Proteinuria (spot urine)
Physical examination and vital signs
Laboratory tests
Haematology/Biochemistry
Lipid profile
Sirolimus trough level
Adverse events and concomitant therapy

^1^Only at baseline and month 24

### Patient follow-up procedures

Study duration for all patients will be 24 months in total, consisting of a 6 months pre-randomisation observational period and 18 months follow up after randomisation (Figure 1). Four main study visits including kidney MRI and 24 h urine collection will take place at baseline, month 6, 12 and 24. Randomisation and inclusion in the study takes place after evaluation of kidney volume growth from baseline to month 6 within 2 weeks after the second MRI. Three additional visits including clinical assessment and blood and urine chemistry will take place at month 9, 15 and 18. Patients in the treatment arm will have four extra visits at week 2, 4, month 1 and 2 after randomisation to allow for blood level monitoring and potential dose adjustment of the study medication. Patients without volume progression during the pre-randomisation period will be followed by MRI for additional 6 months and enrolled if kidney volume enlargement is detectable.

### Study withdrawal

Patients will be censored and withdrawn from follow-up at their request. Patients will also be censored if they are not randomized after the pre-randomization observation period.

### Statistical analysis

Principal analysis will be undertaken using an intention-to-treat approach. A secondary on-treatment analysis will also be performed. The annual percent change in kidney volume will be determined by regressing the log transformed total kidney volume at month 6, 12 and month 24 against time for each patient through the least squares method. Mean annual decline of renal function will be calculated by regressing GFR or creatinine clearance against time. All primary and secondary end point variables will be compared using a two-sided α-level of 0.05. To account for possible baseline imbalances, a secondary analysis will be performed in which comparison of treatment groups for all endpoints will be adjusted for predefined covariates using multiple regressions. The predefined covariates are age, sex, presence of hypertension, medication with angiotensin converting enzyme inhibitors or angiotensin receptor blockers, baseline total kidney volume and percentage kidney growth during the pre-randomisation period.

### Sample size considerations

In a large cohort of ADPKD patients, the mean annual kidney volume growth rate was 5.27% ± 3.92% (SD)[7]. Because patients with lack of progression during the pre-randomisation period will be excluded from our study, we expect to select for a higher progression rate in our study population. Due to a shorter observation interval compared to the mentioned observational study, the standard deviation might be higher. Presuming an annual kidney growth rate of 6% ± 4.75% (SD) in the control group, a sample size of 40 patients per group will have 80% statistical power to detect a 50% relative reduction of kidney volume growth using a two-sided α-level of 0.05. To account for a drop out rate of up to 20%, we plan to randomise a total of 100 patients.

## Discussion

The SUISSE ADPKD study seeks to determine if sirolimus halts kidney volume growth in patients with ADPKD early in the disease course. Study participation is restricted to young patients with preserved kidney function because any effective treatment of ADPKD needs to be started early in the course of the disease to have an impact on long term kidney function. Our trial is the first clinical study addressing this question. If sirolimus treatment can reduce or stop volume growth in patients with maintained kidney function and prior documented kidney volume progression, these ADPKD patients could benefit the most from a treatment with an anti-proliferative agent like sirolimus. Similar studies using specific mTOR inhibitors to halt disease progression have been announced by others (Mayo Clinic, USA; Certican^® ^trial, Novartis, Germany). We anticipate that sirolimus might be an effective therapeutic option for ADPKD patients that are prone to progress to end-stage renal disease.

## Competing interests

The author(s) declare that they have no competing interests.

## Authors' contributions

ALS, ADK and RPW were responsible for identifying the research question and drafting the study protocol. All authors have contributed to the development of the protocol and study design, as members of the study team. ALS and FT were responsible for drafting of this manuscript and all authors provided comments and have read and approved the final version.

## Pre-publication history

The pre-publication history for this paper can be accessed here:


